# Supplementary material for: Vegetation dynamics of abandoned paddy fields and surrounding wetlands in the lower Tumen River Basin, Northeast China
Source: PeerJ. 2019 Apr 8;7:e6704. doi: 10.7717/peerj.6704 (PMC6459177; doi:10.7717/peerj.6704)
Supplement: Table S1 [file peerj-07-6704-s002.docx]

**Supplemental Information**

**Table S1. Proportions of chorological spectrum composition in paddy fields of different ages since abandonment (Ab, years) and in natural wetlands (NAT) in the lower Tumen River, northeast China.**

|  | **Ab＜5** | | **5＜Ab＜15** | | **Ab＞15** | | **NAT** | |
| --- | --- | --- | --- | --- | --- | --- | --- | --- |
| **Chorological spectrum** | **n** | **Percentage (%)** | **n** | **Percentage (%)** | **n** | **Percentage (%)** | **n** | **Percentage (%)** |
| Cosmopolitan | 4 | 6.45 | 2 | 4.88 | 2 | 5.88 | 2 | 5.41 |
| Holarctic | 14 | 22.58 | 12 | 29.27 | 9 | 26.47 | 10 | 27.03 |
| Palaearctic | 25 | 40.32 | 20 | 48.78 | 15 | 44.12 | 17 | 45.95 |
| Holarctic+Ori+Aus+Afro | 6 | 9.68 | 3 | 7.32 | 2 | 5.88 | 2 | 5.41 |
| Palaearctic+Ori+Aus+Afro | 13 | 20.97 | 4 | 9.76 | 6 | 17.65 | 6 | 16.22 |

Abbreviations: Ori = Oriental, Aus = Australian, Afro = Afrotropical.
