# Supplementary material for: Vegetation dynamics of abandoned paddy fields and surrounding wetlands in the lower Tumen River Basin, Northeast China
Source: PeerJ. 2019 Apr 8;7:e6704. doi: 10.7717/peerj.6704 (PMC6459177; doi:10.7717/peerj.6704)
Supplement: Table S2 — and in natural wetlands (NAT). SD: Standard Deviation. [file peerj-07-6704-s003.docx]

**Supplemental Information**

**Table S2.** **Mean of Margalef’s index of species richness (R), Shannon–Wiener** **diversity index (*H*), Simpson’s index (D) and Pilou’s evenness index (J) of plant communities in paddy fields at different times since abandonment** **(Ab, years) and in natural wetlands** **(NAT). SD**: **Standard Deviation**

| **Wetland** | **Margalef’s index of species richness (R)** | | **Shannon-Wiener diversity index (*H*)** | | **Simpon's index(D)** | | **Pielou's eveness index(J)** | |
| --- | --- | --- | --- | --- | --- | --- | --- | --- |
| **Successional stages** | **R** | **±SD** | ***H*** | **±SD** | **D** | **±SD** | **J** | **±SD** |
| **Ab＜5** | 23.00 | **±**1.39 | 0.76 | **±**0.14 | 1.91 | **±**0.38 | 0.61 | **±**0.14 |
| **5＜Ab＜15** | 14.00 | **±**2.43 | 0.66 | **±**0.24 | 1.55 | **±**0.66 | 0.60 | **±**0.20 |
| **Ab＞15** | 15.50 | **±**1.56 | 0.65 | **±**0.14 | 1.46 | **±**0.38 | 0.53 | **±**0.11 |
| **NAT** | 8.82 | **±**1.35 | 0.59 | **±**0.21 | 1.25 | **±**0.47 | 0.60 | **±**0.20 |
