# Supplementary material for: Vegetation dynamics of abandoned paddy fields and surrounding wetlands in the lower Tumen River Basin, Northeast China
Source: PeerJ. 2019 Apr 8;7:e6704. doi: 10.7717/peerj.6704 (PMC6459177; doi:10.7717/peerj.6704)
Supplement: Table S3 [file peerj-07-6704-s004.docx]

| **Matrix fill** | **Ab＜5** | **5＜Ab＜15** | **Ab＞15** |
| --- | --- | --- | --- |
| **S—Similarity** | 6.01% | 6.98% | 11.54% |
| **R—Abundance Replacement** | 83.01% | 79.86% | 77.06% |
| **D—Abundance Difference** | 10.98% | 13.17% | 11.40% |
| **R+D—Beta diversity** | 93.99% | 93.02% | 88.46% |
| **S+R—Abundance Agreement** | 89.02% | 86.83% | 88.60% |
| **S+D—Nestedness** | 15.00% | 17.98% | 21.56% |

**Supplemental Information**

**Table S3. Results of the Simplex SDR analyses for all pairwise comparisons in paddy fields at different times since abandonment (Ab, year)**
