# Supplementary material for: Vegetation dynamics of abandoned paddy fields and surrounding wetlands in the lower Tumen River Basin, Northeast China
Source: PeerJ. 2019 Apr 8;7:e6704. doi: 10.7717/peerj.6704 (PMC6459177; doi:10.7717/peerj.6704)
Supplement: Table S4 [file peerj-07-6704-s005.docx]

**Supplemental Information**

**Table S4: Results of DCA analysis and plot of DCA site scores derived from species percentage coverage.**

| **Axes** | **DCA_1_** | **DCA_2_** | **DCA_3_** | **DCA_4_** |
| --- | --- | --- | --- | --- |
| **Eigenvalues** | 0.8689 | 0.7182 | 0.4747 | 0.3974 |
| **Decorana values** | 0.8878 | 0.6816 | 0.3735 | 0.2899 |
| **Axis lengths** | 6.7989 | 5.7176 | 3.6239 | 2.5878 |

| **Code** | **DCA_1_** | **DCA_2_** | **DCA_3_** | **DCA_4_** |
| --- | --- | --- | --- | --- |
| NAT | -2.3104 | 1.1414 | -0.7424 | -1.4809 |
| NAT | -0.4735 | 2.357 | 0.33 | -0.5404 |
| NAT | -0.278 | 2.6605 | 0.3645 | -0.1091 |
| NAT | 0.3712 | 1.369 | 0.318 | -0.534 |
| NAT | -0.2535 | -1.2959 | 0.0569 | -0.1754 |
| NAT | -2.7511 | -1.6263 | -0.7536 | 0.6762 |
| NAT | -1.9162 | -3.0571 | -0.8797 | -0.7214 |
| NAT | -1.9097 | 0.4174 | 0.9562 | 1.1069 |
| NAT | -2.0645 | 1.0242 | 0.4634 | 0.4755 |
| NAT | -1.3766 | 1.5197 | 0.3151 | -0.1891 |
| NAT | -1.4029 | 0.9014 | 0.1759 | -0.7516 |
| Ab＞15 | -2.3303 | 1.0719 | -0.0503 | 0.4315 |
| Ab＞15 | -1.7958 | 1.1472 | -0.6103 | 1.103 |
| Ab＞15 | -2.1719 | 0.3879 | -0.5519 | -1.0925 |
| Ab＞15 | -1.9266 | -0.4086 | -0.3522 | -0.5082 |
| 5＜Ab＜15 | 2.4272 | 0.1586 | 0.0736 | 0.7088 |
| 5＜Ab＜15 | -0.0442 | -0.137 | -0.6675 | 0.7119 |
| 5＜Ab＜15 | -0.4124 | -1.4385 | 0.2415 | -0.1583 |
| 5＜Ab＜15 | -0.0322 | -1.7808 | -0.2077 | -0.3392 |
| 5＜Ab＜15 | -1.0654 | -1.6389 | 0.2052 | 0.0362 |
| Ab＜5 | 3.3882 | -0.0498 | 1.9291 | -0.0737 |
| Ab＜5 | 4.0478 | 0.0559 | 0.8871 | 0.1823 |
| Ab＜5 | 2.9247 | 0.0778 | 0.3643 | 0.4634 |
| Ab＜5 | 1.8893 | -0.1655 | -0.6745 | 0.9459 |
| Ab＜5 | 3.2904 | 0.4693 | -1.6948 | 0.3393 |
| Ab＜5 | 2.7936 | -0.1368 | 0.9522 | -0.3746 |
