# Supplementary material for: Vegetation dynamics of abandoned paddy fields and surrounding wetlands in the lower Tumen River Basin, Northeast China
Source: PeerJ. 2019 Apr 8;7:e6704. doi: 10.7717/peerj.6704 (PMC6459177; doi:10.7717/peerj.6704)
Supplement: Table S5 [file peerj-07-6704-s006.docx]

**Supplemental Information**

| **Functional groups** | **Ab＜5** | | **5＜ Ab ＜15** | | **Ab＞15** | | **NAT** | |
| --- | --- | --- | --- | --- | --- | --- | --- | --- |
|  | **n** | **Percentage of Species (%)** | **n** | **Percentage of Species (%)** | **n** | **Percentage of Species (%)** | **n** | **Percentage of Species (%)** |
| **Sedge and rush** | 15 | 24.19 | 5 | 12.20 | 6 | 17.65 | 7 | 18.92 |
| **Grass** | 6 | 9.68 | 5 | 12.20 | 6 | 17.65 | 7 | 18.92 |
| **Forb** | 41 | 66.13 | 31 | 75.61 | 22 | 64.71 | 23 | 62.16 |

**Table S5. Proportions of species and coverage of three functional groups in paddy fields at different times since abandonment (Ab, year) and in natural wetland (NAT).**

| **Functional groups** | **Ab＜5** | | **5＜ Ab ＜15** | | **Ab＞15** | | **NAT** | |
| --- | --- | --- | --- | --- | --- | --- | --- | --- |
|  | **Coverage** | **Percentage of Coverage (%)** | **Coverage** | **Percentage of Coverage (%)** | **Coverage** | **Percentage of Coverage (%)** | **Coverage** | **Percentage of Coverage (%)** |
| **Sedge and rush** | 8.17 | 13.58 | 23.10 | 34.33 | 58.23 | 78.98 | 31.00 | 36.47 |
| **Grass** | 12.17 | 20.23 | 8.44 | 12.54 | 8.93 | 12.11 | 31.78 | 37.39 |
| **Forb** | 39.83 | 66.2 | 35.74 | 53.12 | 6.58 | 8.92 | 22.21 | 26.13 |
