# Supplementary material for: Vegetation dynamics of abandoned paddy fields and surrounding wetlands in the lower Tumen River Basin, Northeast China
Source: PeerJ. 2019 Apr 8;7:e6704. doi: 10.7717/peerj.6704 (PMC6459177; doi:10.7717/peerj.6704)
Supplement: Table S6 [file peerj-07-6704-s007.docx]

**Supplemental Information**

**Table S6. Occurrence rate of wetland species and Non-wetland species in paddy fields at different times since abandonment (Ab, year) and in natural wetland (NAT).**

|  | **Wetland species** | | **Non-wetland species** | |
| --- | --- | --- | --- | --- |
| **Successional stages** | **n** | **Percentage (%)** | **n** | **Percentage (%)** |
| **Ab＜5** | 34 | 59.68 | 28 | 40.32 |
| **5＜Ab ＜15** | 24 | 70.73 | 17 | 29.27 |
| **Ab＞15** | 22 | 76.47 | 12 | 23.53 |
| **NAT** | 25 | 94.12 | 12 | 13.51 |
